# A Cell Adhesion-Based Reconstitution Method for Studying Cell Polarity

**DOI:** 10.3389/fcell.2020.598492

**Published:** 2020-10-26

**Authors:** Christopher A. Johnston

**Affiliations:** Department of Biology, University of New Mexico, Albuquerque, NM, United States

**Keywords:** cell polarity, spindle orientation, mitosis, reconstitution, neuroblast

## Abstract

Cell polarity is an evolutionarily conserved process of asymmetric spatial organization within cells and is essential to tissue structure, signal transduction, cell migration, and cell division. The establishment and maintenance of polarity typically involves extensive protein-protein interactions that can be made further intricate by cell cycle-dependent regulation. These aspects can make interpreting phenotypes within traditional *in vivo* genetic systems challenging due to pleiotropic effects in loss-of-function experiments. Minimal reconstitution methods offer investigators the advantage of stricter control of otherwise complex systems and allow for more direct assessment of the role of individual components to the process of interest. Here I provide a detailed protocol for a cell adhesion-based method of inducing cell polarity within non-polarized *Drosophila* S2 cells. This technique is simple, cost effective, moderate throughput, and amenable to RNAi-based loss-of-function studies. The ability to “plug-and-play” genes of interest allows investigators to easily assess the contribution of individual protein domains and post-translational modifications to their function. The system is ideally suited to test not only the requirement of individual components but also their sufficiency, and can provide important insight into the epistatic relationship among multiple components in a protein complex. Although designed for use within *Drosophila* cells, the general premise and protocol should be easily adapted to mammalian cell culture or other systems that may better suit the interests of potential users.

## Introduction

Broadly defined, cell polarity can refer to any asymmetric assembly, organization, or segregation of cellular components. Polarity can involve different subcellular structures, including the cytoskeleton, organelles, and protein complexes at the cell membrane (referred to as “cortical polarity” herein). Cortical polarity involves segregation of protein complexes to discrete regions of the cell cortex, such as apical-basal polarity seen classically in epithelial cells as well as several other diverse cell types (Rodriguez-Boulan and Macara, 2014). One critical function of cortical polarity complexes, which will be the focus of my discussion herein, is directing the orientation of cell division by instructing the positioning of the mitotic spindle. Oriented cell divisions ensure that tissue architecture is properly maintained and also facilitates cell fate acquisition following asymmetric stem cell divisions (Ragkousi and Gibson, 2014). In this paradigm, cortically polarized factors serve as positioning cues for the spindle, which is carried out by microtubule (MT)-associating factors within the polarity complexes. For example, in *Drosophila* neural stems cells (called neuroblasts, NBs) the spindle orientation complex is apically polarized and facilitates spindle positioning through interactions with the Dynein/Dynactin complex and the kinesin protein Khc-73, both direct MT-binding motor proteins (Lu and Johnston, 2013). Although the precise molecular details can differ, similar processes have been identified in epithelial cells of the developing wing disc and ovarium, as well as in the mammalian epidermis, gut epithelia, and developing neocortex (Dewey et al., 2015b; di Pietro et al., 2016). Thus, coupling of cortical polarity with spindle MTs is an evolutionarily conserved mechanism for orienting cell divisions during development.

Much of our knowledge regarding the components involved in this complex process has come from genetic mutants and knockdowns in model organism tissue. While these systems represent ideal models for examining the requirement of a particular gene, and the *in vivo* setting has imminent biological relevance, they are not without potential inherent disadvantages. For example, if the gene of interest is essential for viability of the organism it may not be possible to examine its effects at the desired developmental stage (although this can often be overcome through cell/tissue-specific knockdown strategies). Moreover, loss-of-function in one polarity component can often have deleterious consequences on the expression or localization of one or more other factors, leading to complications in phenotype interpretation. Such outcomes make it challenging to build accurate molecular models and to ascertain the sufficiency of one component or complex. Finally, genetic or functional redundancy in a system can mask otherwise important functions of a single mutated gene.

**FIGURE 1:**
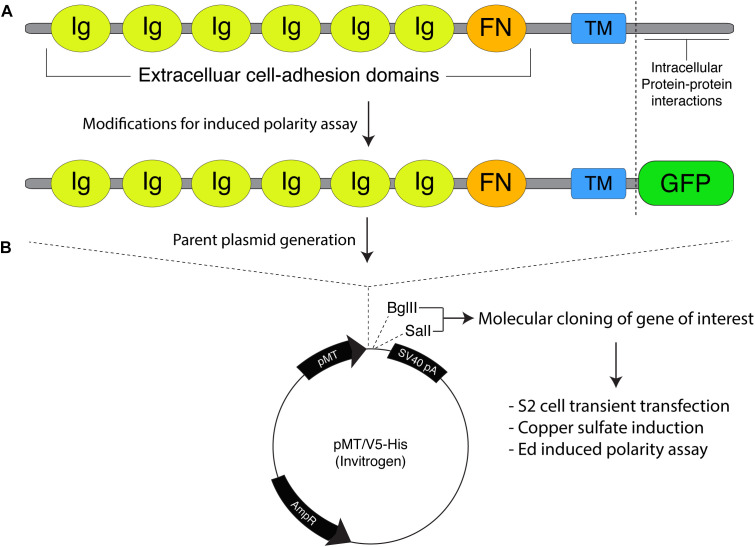
Molecular framework for the Echinoid-based polarity reconstitution system. **(A)** **Top:** Domain architecture of the full-length Ed protein depicts an extracellular region containing several Immunoglobulin (Ig; yellow) and Fibronectin (FN; orange) cell adhesion domains that participate in formation of cell clusters. The transmembrane (TM; blue) region allows for insertion as an integral plasma membrane protein. The C-terminal tail (sequence following vertical dash line) resides intracellularly and is responsible for protein-protein interactions that participate in maintenance of adherens junction function and signaling. **Bottom:** Cloning of Ed for use in the induced polarity assay omits most of the intracellular tail to avoid interactions with known binding partners. This sequence is replaced with an in-frame green fluorescence protein (GFP; green) coding sequence. **(B)** The modified Ed:GFP sequence (with GFP replacing native C-terminal sequence) is cloned into the pMT/V5-His plasmid followed by 5′-*Bgl*II and 3′-*Sal*I cloning sites. Standard molecular cloning can easily generate Ed:GFP fusions to ostensibly any gene or sequence fragment the user wishes to examine. Cells are then transiently transfected with the cloned plasmid, and Ed:GFP fusion proteins are expressed using copper sulfate activation of the pMT promotor (see “Stepwise procedures”).

One way to overcome such drawbacks is through the use of minimal reconstitution systems. “Bottom-up” synthetic approaches offer users a simpler environment to observe complex processes while also providing them with greater experimental control over the construction and operation of the chosen system and its spatial-temporal dynamics (Thery, 2010; Kim et al., 2016; Carbone et al., 2017; Ganzinger and Schwille, 2019). This often results in unique molecular insights that synergize with knowledge obtained from traditional *in vivo* genetic experiments. Such approaches can range from cell-free *in vitro* reconstitutions to fabrication of a minimal network within simple cell culture model and can be used to study a diverse range of cellular processes. Cell polarity is an ideal process to study in a minimal system as it suffers from many of the caveats described above. In recent years, several methods have been developed that offer novel means of reconstituting polarity in non-polar environments (Table 1). Several approaches have also been developed for prokaryotic and simple eukaryotic yeast cells (Vendel et al., 2019). Here, I describe an “induced polarity” assay protocol used in cultured *Drosophila* S2 cells that utilizes the cell adhesion protein, Echinoid (Ed), to reconstitute cortical polarity in these otherwise non-polar cells (Figure 1; Johnston et al., 2009). The method is simple, time- and cost-effective, amenable to RNAi-based loss-of-function analysis, and can be easily adapted for use in other cell culture systems (di Pietro et al., 2017).

**TABLE 1:** Comparison of various *in vitro* methods for reconstituting polarity.

Method	Utility	Notable examples of applications and key discoveries
Cell-free	Most minimal *in vitro* system using specific components selected by user in isolation. Specific concentrations of all components determined by user. Can assess direct interactions among components. Easily adapted to variety of microscopy approaches (e.g., TIRF).	Defining the role of spatial protein concentration gradients in cellular organization and cell division placement site (“Min System”) (Zieske and Schwille, 2014). Determining the molecular organization and function of T-cell immunological synapse (Carbone et al., 2017) [*Also see* (James and Vale, 2012) *for a cell-based method*]. Delineating the role of actin-myosin dynamics in symmetry breaking during polarity initiation (Abu Shah and Keren, 2014).
Micropatterning	User-controlled cell shape dynamics. User-controlled extracellular environment, particularly related to mechano-sensitive signals and cell stiffness. Ability to alter and mimic diverse extracellular matrix patterns.	Determining the role of the extracellular matrix in oriented cell division (Thery et al., 2005). Defining how cell adhesion influences orientation of cell polarity axis (Thery et al., 2006). Identifying a role for cadherins in nuclear and centrosome positioning (Dupin et al., 2009). Defining how extracellular cues and cortical forces influence spindle orientation (Thery et al., 2007).
Optogenetic-based approaches	Highly configurable system to examine structure-function relationships. Ability to test both requirement and sufficiency of specific components. Ability to control both *spatial* and *temporal* aspects of polarization. Adaptable to live-cell imaging.	Identifying organization of cortical force generators and establishing their sufficiency in controlling spindle positioning in human cells (Okumura et al., 2018). Molecular dissection of spindle orientation in *C. elegans* (Fielmich et al., 2018). Probing cell cycle-dependent pathways sufficient to establish polarity in yeast (Witte et al., 2017).
Induced polarity	Similar to optogenetic systems but without specific need for light-sensitive protein fusions. Highly configurable, rapid, and cost-effective. Simple cell shaking protocol for inducing polarity. No requirements for advanced microscope or cell plating technologies.	Discovery of a phosphorylation-dependent Pins/Dlg spindle orientation pathway (Johnston et al., 2009), as well as additional novel regulatory mechanisms for Pins function (Wee et al., 2011; Mauser and Prehoda, 2012; Lu and Prehoda, 2013). Discovery of an actin-mediated spindle orientation pathway involved in Frizzled/Disheveled planar polarity (Johnston et al., 2013), as well as mapping interactions with Mud and Dlg effectors (Segalen et al., 2010; Garcia et al., 2014). Identification of actin regulators involved in spindle orientation in human cells (di Pietro et al., 2017).

**FIGURE 2:**
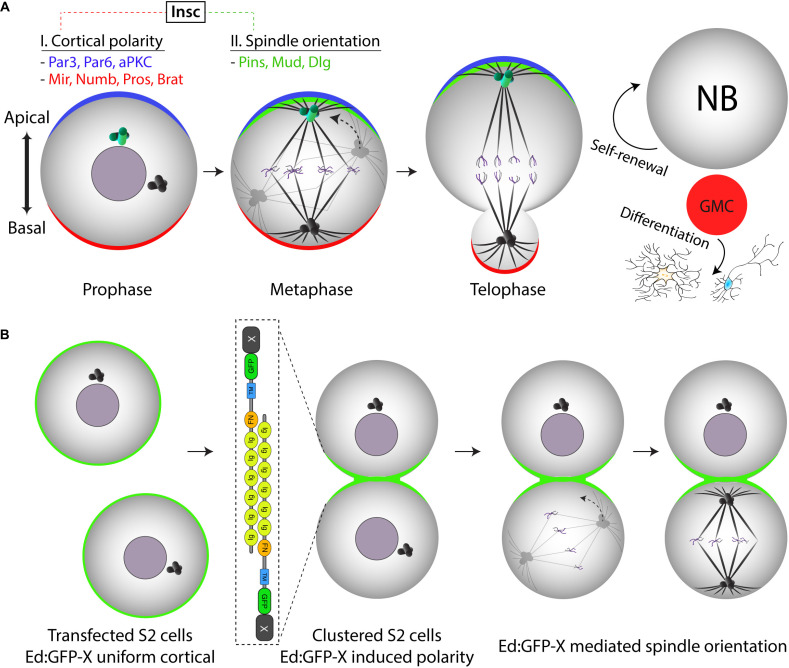
Ed-induced polarity as a minimal reconstitution system to model *Drosophila* neural stem cell spindle orientation. **(A)** *Drosophila* neural stem cells (neuroblasts) establish apical-basal polarity in the early stages of mitosis. Both apical and basal polarity complexes (blue and red, respectively) consist of numerous components connected by a complex network of protein-protein interactions and regulatory relationships. At metaphase, the mitotic spindle aligns along this polarity axis through the activity of the apical Pins/Mud/Dlg spindle orientation complex (green). Mitosis proceeds through an asymmetric cell division that is essential for generating differentiated progeny (via the ganglion mother cell, GMC) while also maintaining the stem cell pool through self-renewal. **(B)** Illustration of how the Ed-induced polarity assay can model Pins-mediated spindle orientation in a minimal reconstituted system (i.e., “X” would represent Pins in this case). Isolated S2 cells initially express an Ed:GFP-X recombinant protein uniformly around the entire cell membrane. Shaking causes collisions that generate cell adhesions wherein cortical Ed:GFP-X proteins concentrate at sites of cell-cell contact within small clusters, the simplest of which is two adhered cells as shown. As cells enter and proceed through mitosis, spindle orientation can be measured relative to the Ed:GFP-X induced crescent similar to how one would with the native Pins crescent in the neuroblast. Note the simplification of this S2 cell system as compared with the complex environment established natively within NBs.

Ed is a key component of the adherens junction complex that, cooperating with DE-cadherin, controls cell-cell adhesion in *Drosophila* through homotypic, intercellular interactions (Wei et al., 2005). In addition to this structural role, Ed also functions in numerous intracellular signaling pathways that contribute to tissue development and dynamics (Shimono et al., 2012). The Ed domain architecture, shown in Figure 1A, is typified by a series of extracellular Immunoglobulin (Ig) and fibronectin (FN) adhesion domains, followed by a transmembrane insertion region and an intracellular C-terminal tail. This short intracellular tail is responsible for protein-protein interactions, most notably with the actin-associated factor Canoe (Afadin in mammals) (Wei et al., 2005; Sawyer et al., 2009). The method presented here capitalizes on this rather simple topology in two principal ways: (1) upon extracellular Ig domain-mediated adhesion, membrane-inserted Ed molecules redistribute specifically to cortical regions at the sites of cell-cell contact, and (2) a specific gene of interest is cloned in-frame with a truncated intracellular tail lacking interactions with other known polarity factors (Figure 1B). Together, these factors lead to the induction of Ed-mediated cortical polarity of ostensibly any protein of interest within non-polar S2 cells (Figure 2). Users can then design experiments that address specific research questions related to polarity or linked processes such as mitotic spindle orientation. This system is highly adaptable and should therefore be useful in reconstituting diverse polarity components that emulate diverse native systems, with *Drosophila* neuroblasts being an ideal example used for illustration and discussion herein (Figure 2A). Expression vectors are designed for simple, “plug-and-play” molecular cloning, and S2 cells are ideally suited for RNA interference (RNAi) loss-of-function screens (Rogers and Rogers, 2008). Overall, this system offers researchers a simple and rapid means of studying cell polarity that can complement studies in traditional genetic systems.

## Materials and Equipment

### Cell Culture

The protocol detailed below is specifically adapted for *Drosophila* Schneider 2 (S2) cells, a cell line originally isolated from late stage embryos thought to be derived from a macrophage-like origin (Schneider, 1972). S2 cells are grown and maintained in Schneider insect media (SIM; Invitrogen) supplemented with 10% fetal bovine serum at 25–29°C without the need for CO_2_ humidification. Standard S2 cell stocks can be purchased from Invitrogen or the Drosophila Genomics Resource Center (DGRC;^1^), which also maintains additional lines stably expressing fluorescent markers for various cell structures (e.g., tubulin). Growth medium can be supplemented with penicillin-streptomycin, although this is not necessary in our experience and should be considered optional. As with other cell culture systems, users should routinely monitor for *Mycoplasma* contamination using standard methods to avoid compromising validity of results (Roth et al., 2020).

### RNAi Preparation

Another distinct advantage of S2 cells is their ability to take up dsRNA directly from media, which is subsequently processed into small interfering sequences avoiding the need to clone and transfect shRNA constructs (Rogers and Rogers, 2008). Rather, dsRNA can be directly transcribed *in vitro* using standard T7 RNA Polymerase with PCR-amplified dsDNA as a template. We typically use the MegaScript T7 synthesis kit (ThermoFisher), although many alternatives are available. Primer design is carried out using the SnapDragon dsRNA Design tool freely available at the Harvard DRSC/TRiP Functional Genomics Resource site^2^. Sequences are chosen based on predicted efficiency and specificity for the selected target, and can be designed in an isoform/spliceform-specific or -universal manner. All primers are then synthesized with 5′ T7 recognition sequences appended (5′-TAATACGACTCACTATAGGG-3′).

### Immunostaining and Microscopy

Cells are plated on glass coverslips treated with poly-lysine to increase their adherence. S2 cells are relatively small (diameter of ∼10 μm) and can be further flattened by coating glass coverslips with the lectin molecule Concanavalin A (ConA). We typically use 12 mm diameter coverslips placed individually into 24-well culture dishes. Fixation conditions should be chosen based on individual experiment outcomes, but paraformaldehyde and ice-cold methanol are the most commonly used agents. Primary antibodies must be adapted to specific needs, and we typically use non-crossreactive secondary antibodies from Jackson ImmunoResearch or Invitrogen conjugated with desired fluorophores. Imaging is conducted using a standard fluorescence microscope, although the use of a confocal system may yield better imaging resolution overall. Representative S2 cell images presented below were acquired on an Olympus IX83 or Zeiss 780 m confocal.

### Data Analysis

Data analysis is performed using the ImageJ software package^3^. For example, spindle positioning can be measured using the angle tool with vertices perpendicular to the midpoint of the Ed crescent and through the spindle midzone. Excel and GraphPad Prism are additional software programs ideally suited for further analysis and graphical output of the data.

## Stepwise Procedures

### Molecular Cloning of Ed Fusion Constructs

Expression of Ed fusion constructs in S2 cells is achieved using the copper inducible metallothionein promotor within the pMT expression vector (Thermo Fisher). Cloning and construction of pMT:Ed plasmids has been previously detailed (Johnston et al., 2009). We have generated plasmids that yield either GFP- or FLAG-tagged versions of the Ed fusion, with the general structure of Ed:GFP-X, where X represents the desired cloned gene of interest (Figure 1). Both plasmids are linearized using 5′-*Bgl*II and 3′-*Sal*I restriction digest, which can be ligated with identically digested inserts or those digested with isocaudameric enzymes such as 5′-*Bam*HI and 3′-*Xho*I. Cloning should be done using standard molecular techniques and verified using Sanger sequencing methods.

### Transient Transfection of S2 Cells

One significant drawback to the use of S2 cells are their relatively low transfection efficiency compared with many other cell culture lines. However, liposome-based transfection reagents are still recommended as standard practice. We typically use Effectene (Qiagen), the protocol for which is described below, although many alternatives exist.

1. Seed S2 cells in 6-well dishes at a density of 1–2 × 10^6^ cells in 2.5 mL of SIM.
2. Prepare Effectene-DNA mixtures according to manufacturer protocol. Cells are typically transfected with a total of 0.5–1 μg of total plasmid DNA.
3. Add transfection mixtures dropwise to respective wells and incubate for 24–48 h.

*Note*: For standard experiments proceed to step 4; for RNAi-based loss-of-function studies see next section for additional experimental steps.

4. Add 0.5 mM copper sulfate and incubate for an additional 24 h prior to proceeding to the Ed assay. Extending the induction time to 48 h may improve expression of larger Ed fusion constructs and should be optimized for individual genes being tested.

### RNAi Preparation and Treatment (For Loss-of-Function Studies)

For studies examining the effects of specific gene knockdown, transfected cells are treated directly with dsRNA.

1. Centrifuge transfected cells (following step 3 above) for 3 min at 1,000 × *g*.
2. Resuspend cell pellets in serum free media (SFM) at 2 × 10^6^ cells/mL.
3. Add 1 mL of resuspended cells to fresh wells of a 6-well dish.
4. Add desired dsRNA to respective wells. In our experience, 10 μg of dsRNA (dissolved in ∼100 μL of RNAase-free water) is sufficient for knockdown of most targets.
5. After 1 h incubation in SFM, add 2.5 mL of SIM and incubate for an additional 3–5 days.

Note: The amount of dsRNA and incubation times may need to be optimized for specific targets. Those listed above are a standard guideline.

6. Add 0.5 mM copper sulfate and incubate for an additional 24 h prior to proceeding to Ed polarity induction.

### Inducing Ed-Based Cell Clusters

**FIGURE 3:**
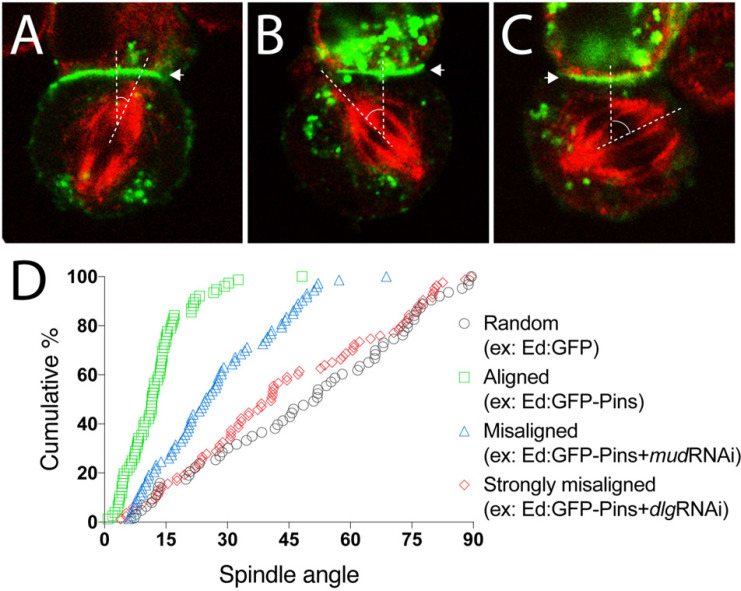
Representative data for spindle orientation in the Ed-induced polarity system. **(A)** Representative image of a mitotic spindle (red) aligned under an Ed:GFP-X crescent (green; arrowhead). Dashed lines show vertices of spindle angle. Such result should be expected for Ed:GFP-Pins, for example. **(B)** Representative image of a misaligned spindle (red) relative to the Ed:GFP-X induced crescent. Dashed lines show vertices of spindle angle. **(C)** Representative image of a strongly misaligned spindle (red) relative to the Ed:GFP-X induced crescent. Dashed lines show vertices of spindle angle. **(D)** Plot of multiple technical and biological replicate spindle angle measurements for the indicated example conditions. Measurements are plotted as a function of the cumulative percentage of cells with an angle at or below a given angle. Random spindle angles measured in cells expressing Ed:GFP alone (gray circles) produces a line along the diagonal, whereas efficient spindle alignment in cells expressing Ed:GFP-Pins (green squares), for example, causes a steep, leftward deflection of this line. As an example of spindle misorientation, knockdown of Mud or Dlg (blue triangles and red diamonds, respectively), two effectors downstream of Pins, lead to different degrees of spindle misalignment.

This protocol hinges upon the ability of transfected cells to form Ed-based adhesions that generate small (2–3 cells) clusters in which cortical Ed is redistributed and concentrated at sites of cell-cell contact (Figures 2B, 3A). These cell clusters can easily be induced using the steps outlined below:

1. Prior to starting the Ed cell adhesion assay, place the desired number of glass coverslips in individual wells of a 24-well dish. Add 0.2 mL of poly-lysine solution and incubate at room temperature for 30–60 min. Aspirate and allow coverslips to dry for 1 h to overnight.

*Note*: To maximize collection of a suitable number of technical replicate measurements (typically ≥30) during imaging, it is recommended to prepare at least three coverslips for each condition.

2. When ready to begin, centrifuge transfected cells for 3 min at 1,000 × *g*.
3. Resuspend cell pellets in 3–4 mL of fresh serum-containing SIM supplemented with 0.5 mM copper sulfate.
4. Add cells to fresh wells of a 6-well dish. Agitate on a platform shaker at ∼250 rpm for 1–3 h at room temperature. Physical collisions among cells induces cell adhesions and cluster formation. While shaking times on the higher end of this scale may be needed for poorly transfected or expressing constructs, users should be cautious that longer shaking times may lead to formation of large clusters (>3 cells) that are typically not suitable for subsequent analysis (see description below).
5. Add 0.5 mL of fresh SIM to each coverslip-containing well of a 24-well dish. Add 0.25 mL of cells from step 4 to each well and allow to incubate at room temperature for 2 h. This incubation allows cells to firmly adhere to coverslips and begin to enter the cell cycle, increasing the percentage of cells in mitosis during subsequent fixation.

### Fixation and Immunostaining

Fixation of S2 cells can be achieved by several methods. Formaldehyde and ice-cold methanol are fixatives typically used by most labs, and below are steps for the simpler formaldehyde-based approach.

1. Aspirate excess SIM from previous incubation step.
2. Gently add 0.5 mL of a 4% paraformaldehyde (PFA) solution in 1× PBS. Allow fixation to proceed for 15 min at room temperature.
3. Remove PFA and perform three quick washes with 0.5 mL of wash buffer (0.1% Triton-X100 in 1× PBS).
4. Following washes, permeabilize cells by incubating in wash buffer for 10 min.

*Note:* Permeabilization conditions may need to be optimized for specific antibodies. Triton X-100 concentrations of 0.1–0.5% and incubation times of 10–20 min should be adequate for most experiments.

5. Add block buffer (wash buffer supplemented with 1% BSA) and incubate for 1 h at room temperature.

*Note*: Users may find that alternative blocking agents, such as 5% goat serum, produce better results and are encourage to optimize blocking conditions for each specific experimental condition.

6. Dilute desired primary antibodies to appropriate concentrations in block buffer. For measuring spindle orientation, we typically use a rat anti-alpha-tubulin at 1:500 dilution (Abcam) to mark the spindle along with a rabbit anti-phosphohistone H3 (PH3) at 1:2000 dilution (ThermoFisher) to mark mitotic DNA. In our experience, natural GFP fluorescence is sufficient for visualizing Ed crescents, although users may consider addition of an anti-GFP antibody as well. Use of Ed:FLAG constructs requires the use of an anti-FLAG primary antibody (e.g., Sigma).
7. Add 0.2 mL primary antibody solution and incubate overnight at 4°C.
8. The next day, remove primary antibody solution and wash cells three times with block buffer.
9. Dilute desired secondary antibodies to appropriate concentrations in block buffer. Keep in mind that, if using Ed:GFP, polarity crescents will necessarily use the 488 nm (e.g., FITC) excitation filter channel. The use of Ed:FLAG allows for additional flexibility when choosing the conjugated fluorophore of the secondary antibody used against the anti-FLAG primary antibody.
10. Add 0.2 mL secondary antibody solution and incubate 1–2 h at room temperature.
11. Remove secondary antibody solution and wash cells three times with wash buffer.
12. Prepare microscope slides by adding a small drop of preferred mounting medium. We typically use VectaShield Hardset, but many alternatives are available. Use of DAPI-containing media should be considered as an alternative method of visualizing DNA, although this will not be specific for mitotic chromosomes.
13. Carefully remove coverslips from wells, dab dry, and mount with cells facing mounting solution.
14. Allow slides to dry for at least 1 h prior to imaging. If imaging on a later day, store slides at 4°C and protected from light.

*Imaging:* Rather than provide a detailed protocol suited for our specific microscopes, below are general steps and important considerations for imaging Ed-based cell clusters in experiments assessing mitotic spindle orientation.

1. After staging the slide on the microscope, begin searching for appropriate cell clusters suitable for imaging. In addition to their relatively low transfection efficiency, S2 cells also suffer from a lower mitotic index compared with many cell culture lines and, therefore, identifying clusters of transfected cells undergoing mitosis is most commonly the bottleneck of the entire protocol. The following criteria are a guideline for selecting candidate cells:
  • Clusters containing 2–3 cells are typically preferred. Clusters containing >3 cells often exhibit excessively wide Ed polarity crescents or multiple, non-continuous crescents per cell, both of which make accurate assessment of spindle positioning challenging or impossible. If cortical polarization of a potential cytoplasmic binding partner of the Ed-fused protein is the intended experimental outcome, rather than spindle orientation, such large clusters may still be suitable, however.
  • The width of the Ed polarity should not exceed ∼30% of the cell circumference. This estimation reflects that of a typical *Drosophila* neuroblast, the native cell originally mimicked by this approach (Figure 2A; Johnston et al., 2009). However, this parameter should be considered *a priori* and adjusted accordingly based on the specific system that the user is attempting to reconstitute or emulate.

*Note:* S2 cells can naturally adhere to one another in an Ed-independent manner. In the case of one transfected cell adhering to a non-transfected cell, the Ed distribution in the transfected cell remains uniform cortical and not polarized. These can be easily distinguished from dual transfected, polarized cells and can be avoided in imaging experiments.

  • For experiments measuring spindle orientation, cells with intact, bipolar spindles should be selected whenever possible to ensure accurate measurements. This criterion should also include cells in which the spindle is parallel to the slide surface and positioned at the approximate cell center. Cells with spindles significantly decentered should be avoided. Users should be especially cautious when selecting cells following RNAi treatment against genes that participate in spindle assembly, where bipolar spindles are not evident. In such cases, DAPI or PH3 staining of the congressed mitotic DNA may aid in analysis.
  • For experiments assessing cell cycle-dependent endpoints, one should include markers of the desired stage for additional selection.
2. Focus the selected cell cluster such that both poles of the mitotic spindle are visible in the chosen focal plane.
3. Adjust other standard parameters (e.g., exposure time, laser intensity, pinhole opening, etc.), then capture and save image. Taking multiple images at evenly spaced z-stack depths may aid in resolving a slightly non-parallel spindle.
4. This process should be repeated for a number of technical replicates suitable for statistical power in data analysis. This is typically achieved with ∼30 replicate images. Biological replicates should then be obtained by repeating the experiment from the beginning of the protocol (e.g., cell transfection).
  • *Note:* as mentioned above, image acquisition is most often the bottleneck of this protocol. To provide users a useful context in this regard, in our experience transfection efficiency of Ed fusions can approach ∼10%, with many of these cells ultimately forming productive clusters (a conservative estimation would be half, thus 5% of cells overall). The mitotic index of S2 cells is typically limited to 1–2%, leading to an overall fraction of cells that are transfected, forming useful polarity clusters, and undergoing mitosis at ∼0.1%. Treatment of cells with RNAi against Cdc27 can improve the mitotic index by accumulating metaphase cells (Goshima et al., 2007).
  • Although not applied to this specific protocol, others have developed more advanced and high-throughput imaging and processing approaches. For example, RNAi-based screens have used automated imaging to assess genes required in spindle assembly (Goshima et al., 2007; Kwon et al., 2008). Khushi et al. (2017) recently developed an automated image analysis software for phenotypic descriptions of mitotic spindles. Finally, utilization of machine learning approaches may offer additional benefits to users with such expertise (Sommer and Gerlich, 2013).

### Data Analysis

The specific method of data analysis should be tailored to the intended outcome metric. For brevity, a brief discussion of spindle orientation analysis is outlined below:

1. Open selected image with ImageJ software.
2. Measure the width of the Ed polarity crescent in mitotic cell and ensure it does not exceed ∼30% of the total cell circumference (or an alternative size determined by the user to best mimic the native system being reconstituted). Use this measurement to identify the center of the crescent.
3. Select the “Angle tool” from the tool selection window. This tool uses a 3-click input, with click-1 and -2 establishing the first vertex and click-3 defining the second of the angle.
4. Click on or directly above the midpoint of the Ed crescent and draw a line perpendicular and toward the cell center, which should be the approximate location of the spindle midzone. Click a second time here and then extend a line through the middle of the spindle toward the pole closer to either side of the Ed crescent. Click a third time to finish defining the desired angle and use the “Analyze > Measure” command. A separate window will appear with the measured angle listed.

*Note:* In this standard assay one cannot discriminate between the two centrosomes as with neuroblasts and other cell types (Roubinet and Cabernard, 2014). As such, the spindle pole proximal to the Ed crescent should be selected and the measured angle should never exceed 90°. In other words, a perfectly aligned spindle would measure 0°, and a perfectly misaligned spindle would measure 90°.

5. Repeat this process for all technical replicates.
6. Methods for plotting the data are at the user’s discretion. Our style of choice is to plot the cumulative percentage of cells with spindle measuring ≤ a given angle (*y*-axis) as a function of spindle angle (*x*-axis). These calculations can be made in Excel using an ascending rank order of angles measured in steps 4–5.
7. Graphical representation is also a personal choice. Our program of choice is GraphPad Prism (Figure 3D).

## Anticipated Results

We and others have used this procedure to both confirm *in vivo* results in a minimal system as well as a discovery tool for novel components of cell polarity and mitotic spindle orientation (Johnston et al., 2009, 2013; Dewey et al., 2015a; di Pietro et al., 2017). Presented here are representative results users can anticipate for a typical experiment examining known components of a conserved spindle positioning complex, that being the polarity protein Partner of Inscuteable (Pins; known as LGN in humans) and its direct binding partners Mushroom body defect (Mud; known as NuMA in humans) and Discs large (Dlg; also called SAP97 in humans). Cortically tethered Pins directs spindle orientation through these binding partners’ association with the Dynein/Dynactin and KHC-73 kinesin microtubule motor complex complexes, respectively (Siegrist and Doe, 2005; Bowman et al., 2006; Siller et al., 2006; Johnston et al., 2009; Bergstralh et al., 2013). Loss-of-function in each of these components leads to spindle orientation defects in *Drosophila* neuroblasts as well as several other diverse cell types and organisms (Figure 2A). Untangling the underlying molecular mechanisms was facilitated greatly by the reconstitution system described herein (Johnston et al., 2009, 2013).

Using Ed:GFP alone as a negative control, one should anticipate spindle orientation measurements evenly and randomly distributed between 0–90°. Plotting such results as cumulative percent of measurements as a function of spindle angle should produce a nearly linear line through the diagonal of the plot (Figure 3D). Additionally, calculating the mean of all measurements should yield ∼45° with a relatively large deviation due to the expected randomness of measurements.

When examining Ed:GFP-Pins, as a prototypical example, spindle orientation is biased toward the Ed-induced Pins polarity crescent center, similar to natively cortical Pins in neuroblasts (Figure 2), thus skewing measurements to small angles. Graphically, this leads to a steep and leftward shifted curve compared to the Ed:GFP alone as a greater percentage of cells accumulate within acute angles and few, if any, cells display grossly misoriented spindles with large angle measurements (Figures 3A,D). The resulting average spindle angle is ∼10° in a typical Ed:GFP-Pins experiment. In the event that a predicted spindle orientation factor does not produce these results, users should consider several possibilities. If the Ed-fused component being examined requires association with additional factors (e.g., Pins requires several downstream factors listed above) it is possible that S2 cells do not express these additionally required proteins. Several additional cultured *Drosophila* cells lines exist, are readily available for purchase^4^, and transcriptomic analyses have shown differences in gene expression among them that might help identify an alternative system in this scenario (Cherbas et al., 2011). Similarly, if the component in question requires posttranslational modification, such as phosphorylation, it is possible that S2 cells do not express the necessary enzyme or signaling pathway. If the Ed-fused component requires specific structural features (e.g., oligomerization through sequences such as coiled-coil domains) it is possible that fusion to the Ed protein or its artificial tethering at the cell membrane does not allow such topology or assembly. Finally, it is possible that the component being tested is simply not sufficient to orient the spindle, even if cells express other binding factors.

Once a specific component has been found to orient mitotic spindles as an Ed fusion, users can design simple RNAi-mediated loss-of-function experiments to identify additional factors required for its function. For example, treatment with RNAi against Mud reduces the efficiency of spindle orientation to Ed:GFP-Pins crescents (Figures 3B,D), a result similar to its established role in neuroblasts (Bowman et al., 2006; Siller et al., 2006). The observation that Ed:GFP-Pins retained partial activity following Mud knockdown, which was not initially anticipated, helped identify Dlg as a second direct component in Pins-mediated spindle orientation (Johnston et al., 2009). Knockdown of Dlg, in contrast, leads to a strongly misaligned spindle orientation phenotype in the Ed:GFP-Pins S2 cell system (Figures 3C,D). In addition to RNAi-mediated knockdown experiments, the relative ease with which users can generate point mutations, domain deletions, and chimeric constructs with standard molecular techniques allows for additional means toward rapid and precise dissection of the underlying molecular mechanisms and evolutionary conservation of a gene of interest. Collectively, these experiments can help identify novel spindle orientation pathways and their components as well as establish the epistatic relationship among previously known components, both of which can aid significantly in discerning molecular mechanisms of spindle positioning.

## Discussion

Cell polarity and processes such as mitotic spindle orientation linked closely with it are complex cellular events involving numerous components and regulatory inputs. Understanding how these components cooperate to control these processes and the underlying molecular mechanisms controlling their function can present challenges to traditional genetic approaches using *in vivo* model systems. Minimal reconstitution systems have proven powerful tools in addressing these drawbacks and provide alternative systems to dissect these molecular complexities. The protocol described here offers users a rapid and cost-effective method for inducing polarity within a simple, non-polar cell line that should help continue resolving unanswered questions in this conserved and essential cellular process. Although specifically designed for *Drosophila* cells, the protocol can be adapted for use in mammalian systems. For example, a recent study used a similar approach in HeLa cells, a mainstay in mitosis research in human cells (di Pietro et al., 2017). Such adaptations will help further define mechanisms of spindle positioning as well as in comparisons among evolutionarily related genes.

It should be discussed that this approach, like most, is not without limitations. First, as mentioned above, perhaps most substantive is the potential bottleneck one may encounter during imaging. Compounding issues of S2 cell transfection efficiency and mitotic index can sometimes limit the throughput of data collection. However, this has not been a terminal issue, as we and others have used this protocol in numerous studies that required extensive conditions to be tested using it as the primary method. Users are, nevertheless, cautioned to anticipate such limits when planning experiments. Second, studies using this protocol to investigate cortical polarity have mostly focused on how Ed-fused proteins affect localization of cytoplasmic proteins. As such, its application to studying effects on other integral membrane proteins has not yet been well-established. Finally, the system obviously requires first identifying an Ed fusion that is sufficient to evoke a measureable effect (e.g., Ed:Pins orienting the spindle). While this allows subsequent dissection of the molecular aspects of its function, the ability to specifically examine Ed fusions of its downstream effectors may not be possible if their function is required but not sufficient. For example, Pins functions through two effectors, Mud and Dlg, and whereas Dlg is sufficient to induce a partial orientation of spindles in this system, Mud is not. Thus, the Ed system was ideal to probe the mechanism of Dlg function further, but was limited in the information it could provide for Mud activity (Johnston et al., 2009).

## Data Availability Statement

The original contributions presented in the study are included in the article/supplementary material, further inquiries can be directed to the corresponding author/s.

## Author Contributions

CJ conceptualized and wrote the manuscript, generated the results, and prepared the figures and table.

## Conflict of Interest

The author declares that the research was conducted in the absence of any commercial or financial relationships that could be construed as a potential conflict of interest.

## Footnotes

1 https://dgrc.bio.indiana.edu/Home

2 https://www.flyrnai.org/snapdragon

3 https://imagej.nih.gov/ij/download.html

4 https://dgrc.bio.indiana.edu/cells/Catalog

## References

[B1] Abu Shah E. Keren K. (2014). Symmetry breaking in reconstituted actin cortices. *eLife* 3:e01433. 10.7554/eLife.01433 24843007 PMC3996625

[B2] Bergstralh D. T. Lovegrove H. E. St Johnston D. (2013). Discs large links spindle orientation to apical-basal polarity in *Drosophila epithelia*. *Curr. Biol.* 23 1707–1712. 10.1016/j.cub.2013.07.017 23891112 PMC3770898

[B3] Bowman S. K. Neumuller R. A. Novatchkova M. Du Q. Knoblich J. A. (2006). The *Drosophila* NuMA Homolog Mud regulates spindle orientation in asymmetric cell division. *Dev. Cell.* 10 731–742. 10.1016/j.devcel.2006.05.005 16740476

[B4] Carbone C. B. Kern N. Fernandes R. A. Hui E. Su X. Garcia K. C. (2017). In vitro reconstitution of T cell receptor-mediated segregation of the CD45 phosphatase. *Proc. Natl. Acad. Sci. U.S.A.* 114 E9338–E9345. 10.1073/pnas.1710358114 29042512 PMC5676914

[B5] Cherbas L. Willingham A. Zhang D. Yang L. Zou Y. Eads B. D. (2011). The transcriptional diversity of 25 *Drosophila* cell lines. *Genome Res.* 21 301–314. 10.1101/gr.112961.110 21177962 PMC3032933

[B6] Dewey E. B. Sanchez D. Johnston C. A. (2015a). Warts phosphorylates mud to promote pins-mediated mitotic spindle orientation in *Drosophila*, independent of yorkie. *Curr. Biol.* 25 2751–2762. 10.1016/j.cub.2015.09.025 26592339 PMC4660254

[B7] Dewey E. B. Taylor D. T. Johnston C. A. (2015b). Cell fate decision making through oriented cell division. *J. Dev. Biol.* 3 129–157. 10.3390/jdb3040129 26844213 PMC4734650

[B8] di Pietro F. Echard A. Morin X. (2016). Regulation of mitotic spindle orientation: an integrated view. *EMBO Rep.* 17 1106–1130. 10.15252/embr.201642292 27432284 PMC4967962

[B9] di Pietro F. Valon L. Li Y. Goiame R. Genovesio A. Morin X. (2017). An RNAi screen in a novel model of oriented divisions identifies the actin-capping protein Z beta as an essential regulator of spindle orientation. *Curr. Biol.* 27 2452.e8–2464.e8. 10.1016/j.cub.2017.06.055 28803871

[B10] Dupin I. Camand E. Etienne-Manneville S. (2009). Classical cadherins control nucleus and centrosome position and cell polarity. *J. Cell Biol.* 185 779–786. 10.1083/jcb.200812034 19487453 PMC2711586

[B11] Fielmich L. E. Schmidt R. Dickinson D. J. Goldstein B. Akhmanova A. van den Heuvel S. (2018). Optogenetic dissection of mitotic spindle positioning in vivo. *eLife* 7:e38198. 10.7554/eLife.38198 30109984 PMC6214656

[B12] Ganzinger K. A. Schwille P. (2019). More from less - bottom-up reconstitution of cell biology. *J. Cell Sci.* 132:jcs227488. 10.1242/jcs.227488 30718262

[B13] Garcia J. D. Dewey E. B. Johnston C. A. (2014). Dishevelled binds the Discs large ‘Hook’ domain to activate GukHolder-dependent spindle positioning in *Drosophila*. *PLoS One* 9:e114235. 10.1371/journal.pone.0114235 25461409 PMC4252473

[B14] Goshima G. Wollman R. Goodwin S. S. Zhang N. Scholey J. M. Vale R. D. (2007). Genes required for mitotic spindle assembly in *Drosophila* S2 cells. *Science* 316 417–421. 10.1126/science.1141314 17412918 PMC2837481

[B15] James J. R. Vale R. D. (2012). Biophysical mechanism of T-cell receptor triggering in a reconstituted system. *Nature* 487 64–69. 10.1038/nature11220 22763440 PMC3393772

[B16] Johnston C. A. Hirono K. Prehoda K. E. Doe C. Q. (2009). Identification of an Aurora-A/PinsLINKER/Dlg spindle orientation pathway using induced cell polarity in S2 cells. *Cell* 138 1150–1163. 10.1016/j.cell.2009.07.041 19766567 PMC2789599

[B17] Johnston C. A. Manning L. Lu M. S. Golub O. Doe C. Q. Prehoda K. E. (2013). Formin-mediated actin polymerization cooperates with Mushroom body defect (Mud)-Dynein during Frizzled-Dishevelled spindle orientation. *J. Cell Sci.* 126(Pt 19), 4436–4444. 10.1242/jcs.129544 23868974 PMC3784822

[B18] Khushi M. Dean I. M. Teber E. T. Chircop M. Arthur J. W. Flores-Rodriguez N. (2017). Automated classification and characterization of the mitotic spindle following knockdown of a mitosis-related protein. *BMC Bioinformatics* 18(Suppl. 16):566. 10.1186/s12859-017-1966-4 29297284 PMC5751558

[B19] Kim A. K. DeRose R. Ueno T. Lin B. Komatsu T. Nakamura H. (2016). Toward total synthesis of cell function: reconstituting cell dynamics with synthetic biology. *Sci. Signal.* 9:re1. 10.1126/scisignal.aac4779 26861045

[B20] Kwon M. Godinho S. A. Chandhok N. S. Ganem N. J. Azioune A. Thery M. (2008). Mechanisms to suppress multipolar divisions in cancer cells with extra centrosomes. *Genes Dev.* 22 2189–2203. 10.1101/gad.1700908 18662975 PMC2518815

[B21] Lu M. S. Johnston C. A. (2013). Molecular pathways regulating mitotic spindle orientation in animal cells. *Development* 140 1843–1856. 10.1242/dev.087627 23571210 PMC3631962

[B22] Lu M. S. Prehoda K. E. A. (2013). NudE/14-3-3 pathway coordinates dynein and the kinesin Khc73 to position the mitotic spindle. *Dev. Cell* 26 369–380. 10.1016/j.devcel.2013.07.021 23987511 PMC3786870

[B23] Mauser J. F. Prehoda K. E. (2012). Inscuteable regulates the Pins-Mud spindle orientation pathway. *PLoS One* 7:e29611. 10.1371/journal.pone.0029611 22253744 PMC3254608

[B24] Okumura M. Natsume T. Kanemaki M. T. Kiyomitsu T. (2018). Dynein-Dynactin-NuMA clusters generate cortical spindle-pulling forces as a multi-arm ensemble. *eLife* 7:e36559. 10.7554/eLife.36559 29848445 PMC6037482

[B25] Ragkousi K. Gibson M. C. (2014). Cell division and the maintenance of epithelial order. *J. Cell Biol.* 207 181–188. 10.1083/jcb.201408044 25349258 PMC4210436

[B26] Rodriguez-Boulan E. Macara I. G. (2014). Organization and execution of the epithelial polarity programme. *Nat. Rev. Mol. Cell Biol.* 15 225–242. 10.1038/nrm3775 24651541 PMC4211427

[B27] Rogers S. L. Rogers G. C. (2008). Culture of *Drosophila* S2 cells and their use for RNAi-mediated loss-of-function studies and immunofluorescence microscopy. *Nat. Protoc.* 3 606–611. 10.1038/nprot.2008.18 18388942

[B28] Roth J. S. Lee T. D. Cheff D. M. Gosztyla M. L. Asawa R. R. Danchik C. (2020). Keeping it clean: the cell culture quality control experience at the national center for advancing translational sciences. *SLAS Discov.* 25 491–497. 10.1177/2472555220911451 32233736 PMC8506661

[B29] Roubinet C. Cabernard C. (2014). Control of asymmetric cell division. *Curr. Opin. Cell Biol.* 31 84–91. 10.1016/j.ceb.2014.09.005 25264944

[B30] Sawyer J. K. Harris N. J. Slep K. C. Gaul U. Peifer M. (2009). The *Drosophila* afadin homologue Canoe regulates linkage of the actin cytoskeleton to adherens junctions during apical constriction. *J. Cell Biol.* 186 57–73. 10.1083/jcb.200904001 19596848 PMC2712996

[B31] Schneider I. (1972). Cell lines derived from late embryonic stages of *Drosophila melanogaster*. *J. Embryol. Exp. Morphol.* 27 353–365. 4625067

[B32] Segalen M. Johnston C. A. Martin C. A. Dumortier J. G. Prehoda K. E. David N. B. (2010). The Fz-Dsh planar cell polarity pathway induces oriented cell division via Mud/NuMA in *Drosophila* and *Zebrafish*. *Dev. Cell.* 19 740–752. 10.1016/j.devcel.2010.10.004 21074723 PMC3008569

[B33] Shimono Y. Rikitake Y. Mandai K. Mori M. Takai Y. (2012). Immunoglobulin superfamily receptors and adherens junctions. *Subcell. Biochem.* 60 137–170. 10.1007/978-94-007-4186-7_7 22674071

[B34] Siegrist S. E. Doe C. Q. (2005). Microtubule-induced Pins/Galphai cortical polarity in *Drosophila neuroblasts*. *Cell* 123 1323–1335. 10.1016/j.cell.2005.09.043 16377571

[B35] Siller K. H. Cabernard C. Doe C. Q. (2006). The NuMA-related Mud protein binds Pins and regulates spindle orientation in *Drosophila neuroblasts*. *Nat. Cell Biol.* 8 594–600. 10.1038/ncb1412 16648843

[B36] Sommer C. Gerlich D. W. (2013). Machine learning in cell biology - teaching computers to recognize phenotypes. *J. Cell Sci.* 126(Pt 24), 5529–5539. 10.1242/jcs.123604 24259662

[B37] Thery M. (2010). Micropatterning as a tool to decipher cell morphogenesis and functions. *J. Cell Sci.* 123(Pt 24), 4201–4213. 10.1242/jcs.075150 21123618

[B38] Thery M. Jimenez-Dalmaroni A. Racine V. Bornens M. Julicher F. (2007). Experimental and theoretical study of mitotic spindle orientation. *Nature* 447 493–496. 10.1038/nature05786 17495931

[B39] Thery M. Racine V. Pepin A. Piel M. Chen Y. Sibarita J. B. (2005). The extracellular matrix guides the orientation of the cell division axis. *Nat. Cell Biol.* 7 947–953. 10.1038/ncb1307 16179950

[B40] Thery M. Racine V. Piel M. Pepin A. Dimitrov A. Chen Y. (2006). Anisotropy of cell adhesive microenvironment governs cell internal organization and orientation of polarity. *Proc. Natl. Acad. Sci. U.S.A.* 103 19771–19776. 10.1073/pnas.0609267103 17179050 PMC1750916

[B41] Vendel K. J. A. Tschirpke S. Shamsi F. Dogterom M. Laan L. (2019). Minimal in vitro systems shed light on cell polarity. *J. Cell Sci.* 132:jcs217554. 10.1242/jcs.217554 30700498

[B42] Wee B. Johnston C. A. Prehoda K. E. Doe C. Q. (2011). Canoe binds RanGTP to promote Pins(TPR)/Mud-mediated spindle orientation. *J. Cell Biol.* 195 369–376. 10.1083/jcb.201102130 22024168 PMC3206335

[B43] Wei S. Y. Escudero L. M. Yu F. Chang L. H. Chen L. Y. Ho Y. H. (2005). Echinoid is a component of adherens junctions that cooperates with DE-Cadherin to mediate cell adhesion. *Dev. Cell.* 8 493–504. 10.1016/j.devcel.2005.03.015 15809032

[B44] Witte K. Strickland D. Glotzer M. (2017). Cell cycle entry triggers a switch between two modes of Cdc42 activation during yeast polarization. *eLife* 6:e26722. 10.7554/eLife.26722 28682236 PMC5536948

[B45] Zieske K. Schwille P. (2014). Reconstitution of self-organizing protein gradients as spatial cues in cell-free systems. *eLife* 3:e03949. 10.7554/eLife.03949 25271375 PMC4215534

