# Corrigendum: A Cell Adhesion-Based Reconstitution Method for Studying Cell Polarity

**DOI:** 10.3389/fcell.2021.705599

**Published:** 2021-06-22

**Authors:** Christopher A. Johnston

**Affiliations:** Department of Biology, University of New Mexico, Albuquerque, NM, United States

**Keywords:** cell polarity, spindle orientation, mitosis, reconstitution, neuroblast

In the original article, there was an error. The restriction enzymes that are to be used when cloning target genes of interest into the Ed-modified pMT/V5-His plasmid were incorrectly described as “5'-*Bam*HI and 3'-*Xho*I” instead of “5'-*Bgl*II and 3'-*Sal*I.”

A correction has been made to the legend and image of Figure 1B as published and the corrected Figure 1 appears below.

**Figure 1:**
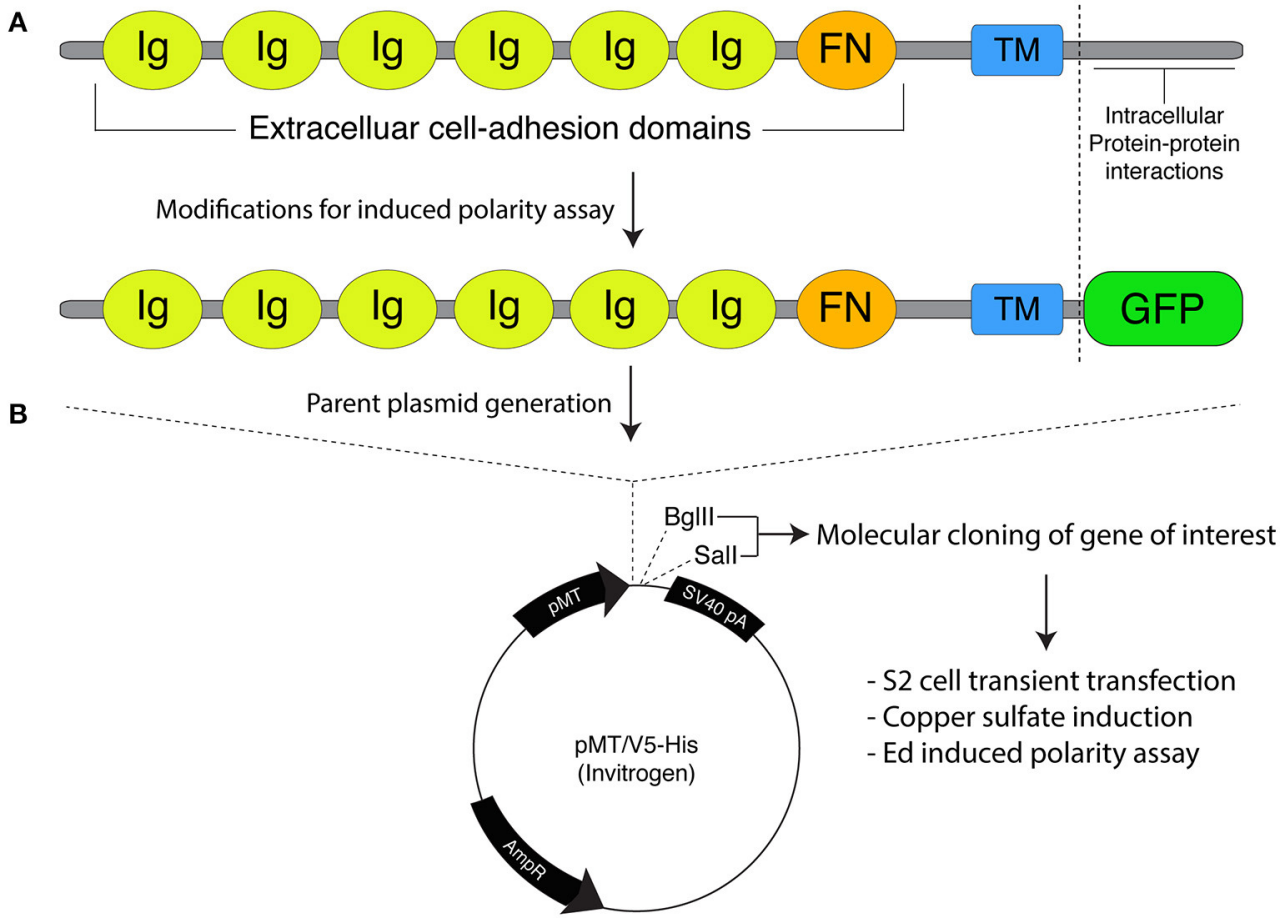
Molecular framework for the Echinoid-based polarity reconstitution system. **(A) Top:** Domain architecture of the full-length Ed protein depicts an extracellular region containing several Immunoglobulin (Ig; yellow) and Fibronectin (FN; orange) cell adhesion domains that participate in formation of cell clusters. The transmembrane (TM; blue) region allows for insertion as an integral plasma membrane protein. The C-terminal tail (sequence following vertical dash line) resides intracellularly and is responsible for protein-protein interactions that participate in maintenance of adherens junction function and signaling. **Bottom:** Cloning of Ed for use in the induced polarity assay omits most of the intracellular tail to avoid interactions with known binding partners. This sequence is replaced with an in-frame green fluorescence protein (GFP; green) coding sequence. **(B)** The modified Ed:GFP sequence (with GFP replacing native C-terminal sequence) is cloned into the pMT/V5-His plasmid followed by 5′-*Bgl*II and 3′-*Sal*I cloning sites. Standard molecular cloning can easily generate Ed:GFP fusions to ostensibly any gene or sequence fragment the user wishes to examine. Cells are then transiently transfected with the cloned plasmid, and Ed:GFP fusion proteins are expressed using copper sulfate activation of the pMT promotor (see “Stepwise procedures”).

A correction has been made to **Stepwise Procedures, Molecular Cloning of Ed Fusion Constructs**:

“Expression of Ed fusion constructs in S2 cells is achieved using the copper inducible metallothionein promotor within the pMT expression vector (Thermo Fisher). Cloning and construction of pMT:Ed plasmids has been previously detailed (Johnston et al., 2009). We have generated plasmids that yield either GFP- or FLAG-tagged versions of the Ed fusion, with the general structure of Ed:GFP-X, where X represents the desired cloned gene of interest (Figure 1). Both plasmids are linearized using 5′-*Bgl*II and 3′-*Sal*I restriction digest, which can be ligated with identically digested inserts or those digested with isocaudameric enzymes such as 5′-*Bam*HI and 3′-*Xho*I. Cloning should be done using standard molecular techniques and verified using Sanger sequencing methods.”

The author apologizes for this error and state that this does not change the scientific conclusions of the article in any way. The original article has been updated.
